# Drug-drug interaction of acetaminophen and roxithromycin with the cocktail of cytochrome P450 and hepatotoxicity in rats

**DOI:** 10.7150/ijms.38527

**Published:** 2020-02-04

**Authors:** Xiang Liu, Chen Chen, Xiaoying Zhang

**Affiliations:** 1 Chinese-German Joint Laboratory for Natural Product Research, College of Biological Science and Engineering, Shaanxi University of Technology, Hanzhong 723001, P.R. China.; 2 Centre of Molecular and Environmental Biology University of Minho, Department of Biology, Campus de Gualtar, Braga, 4710-057, Portugal.

**Keywords:** acetaminophen (APAP), roxithromycin (ROX), cytochrome P450 (CYP450), drug-drug interactions (DDIs), hepatotoxicity

## Abstract

Acetaminophen (APAP) and roxithromycin (ROX) are often used in combination in clinical practice. To evaluate their drug-drug interactions (DDIs) and the hepatotoxicity of co-administration, rats were randomly separated into four groups: Control, APAP (50 mg/kg), ROX (5.5 mg/kg) and APAP-ROX (50 mg/kg and 5.5 mg/kg, respectively). The pharmacokinetic parameters between APAP and ROX were assayed by HPLC, and a cocktail method was used to evaluate the activities of cytochrome (CYP) 450. The liver microsome CYP2E1 protein was detected using Western blot. The levels of plasma parameters, mRNA levels of inflammatory factors (TNF-α, INF-γ, VCAM-1, CXCL-1 and STAT-3) and antioxidant factors (Nrf-2, GSTA, GCLC-1, HO-1 and NQO1) were determined using real-time PCR, along with the observation on histopathological changes in the liver tissue. APAP and ROX co-treatment significantly increased CYP2E1 activity, decreased CYP2D6 activity and prolonged APAP and ROX clearance. Co-treatment increased mRNA expressions of TNF-α, NQO1 and MDA while decreasing GPX and SOD levels. Histopathological evidence showed the changes of liver tissues in terms of structure, size and tight arrangement. This study confirmed that a combination of APAP and ROX inhibited APAP metabolism and that the peak concentration of ROX was delayed. The resulting high level of CYP2E1 may induce oxidative stress and cause liver damage.

## Introduction

Drug combinations may enhance the therapeutic effect of individual drugs, while drug-drug interactions (DDIs) are generally unfavourable. Co-administration may alter the drug-handling capacity of each drug, leading to modified clearance, enhanced side effects and failure of drug therapy 1. The primary focus in pharmacokinetic DDIs is the cytochrome P450 (CYP450) enzyme family. This is because of their promiscuity and prevalence in the metabolism of many drugs and xenobiotics, as many chemicals have been identified as inhibitors or inducers of CYP450, majorly in the liver, including CYP2E1, CYP1A2, CYP2D6 and CYP3A4 2. Inhibition or induction of CYP450 enzymes has been recognised as the determinant of metabolic drug interactions and as significantly increasing the risk of serious adverse reactions or curative failure 3.

Drug metabolites may be an important factor in hepatotoxicity. Recent studies have shown that the incidence of drug-induced liver injury (DILI) in China is at least 23.80/100,000 4, which is particularly striking when compared to the incident rate of 2.7/100,000 in the US and the rate ranging from 1/100,000 to 20/100,000 in Europe 5, 6. The most important drugs causing liver injury in China are various health products and traditional Chinese medicine (26.81%), anti-TB drugs (21.99%), anticancer drugs or immune adjustment agents (8.34%) 4.

Acetaminophen (APAP) is a widely used analgesic and antipyretic drug 7. Roxithromycin (ROX) is used for the treatment of upper respiratory tract infections, as well as skin and soft tissue infections caused by bacteria 8. This study aimed to investigate the drug-drug interactions of a mixture of APAP and ROX on hepatotoxicity by evaluating CYP450.

## Materials and Methods

### Animals and treatments

A total of 40 eight-week-old inbred Sprague-Dawley (SD) female rats (260 ± 20g) were purchased from College of Medicine, Xi'an Jiaotong University (Xi'an, China). All rats were maintained in a conventional sanitary facility, with the required consistent temperature and relative humidity. All experimental animal protocols were reviewed and approved by the institutional Ethics Committee for the use of laboratory animals.

### APAP and ROX on CYP450 activities

Rats were randomly divided into four groups of five (n=5): normal control (NC, 0.9% physiological saline); APAP (50 mg/kg), ROX (5.5 mg/kg) and APAP-ROX (50 mg/kg APAP and 5.5 mg/kg ROX). The drugs were intragastrically administrated twice per day to each group for three days. After consecutive oral administration for three days, a cocktail solution at a dose of 5 mL/kg, which contained chlorzoxazone (20 mg/kg) in a CMC-Na solution, was administered orally to each group. Pre-dose was started at 0 h, followed 0.167, 0.25, 0.5, 1, 2, 4, 6, 12, 24 and 36 h. The blood samples were collected and centrifuged at 5939 g for 10 min. Plasma samples were collected and stored at -80°C until use.

### Liver injury investigation on rats

As in the CYP450 effects procedure, the rats were randomly divided into four groups of five (n=5): normal control (NC, 0.9% physiological saline), APAP (50 mg/kg), ROX (5.5 mg/kg) and APAP-ROX (50 mg/kg APAP and 5.5 mg/kg ROX). The drugs were intragastrically administrated twice per day to each group for three days. The animals were weighed and sacrificed to collect blood and liver tissue samples. The serum was separated from the blood samples by centrifugation at 900 g and 4°C for 10 min and stored at -20°C until further analysis.

### Chromatographic conditions

Analyses were performed with a 1260 series liquid chromatographer (Agilent, Santa Clara, USA) equipped with a quaternary pump, an autosampler and a thermostatted column compartment (Phenomenex, Torrance, USA). Chromatographic separation was achieved on a 100 mm × 4.6 mm, 3.5 μm particle Agilent Zorbax Eclipse Plus-C18 columns at 25°C. The mobile phase consisted of 0.1% formic acid in water (A) and acetonitrile (B) in elution as follows: A:B (85:15). The detection wavelength was 249 nm for APAP and 0.1mol/L ammonium dihydrogen phosphate (pH6.5A) and acetonitrile (B) in elution as follows: A:B (65:35). The detection wavelength was 230 nm for ROX, the flow rate was 0.8 mL/min, the column temperature was 25°C, and the typical injection volume was 20 μL.

### HPLC sample preparation

All plasma samples were homogenised with acetonitrile. After centrifugation at 5939 g and 4°C for 10 min, the supernatant was stored at 4°C for 24 h, and centrifuged again under the same conditions. The supernatant was filtered through a 0.22 μm filter, and 20 μL of the sample filter liquid was injected into the HPLC system for analysis. The standard curve consisted of samples containing 3.125, 6.25, 12.5, 25 and 50 μg/mL of APAP and 12.5, 25, 50, 100, 125 and 250 μg/mL of ROX.

### Pharmacokinetic analysis

Model fitting and evaluation of the pharmacokinetic parameters were carried out using Drug and Statistics 2.0 (DAS); pharmacokinetic parameters were determined using the non-compartmental method based on the statistical moment theory.

### Serum ALT and AST assays

Enzymatic activities of serum alanine aminotransferase (ALT) and aspartate aminotransferase (AST) were estimated spectrophotometrically using commercial diagnostic kits (Jiancheng Institute of Biotechnology, Nanjing, China).

### Hepatic MDA, SOD and GPX determinations

Frozen liver tissues were thawed and homogenised in ice-cold PBS. The homogenate was centrifuged at 900 g for 10 min at 4°C, and the supernatants were assayed for malondialdehyde (MDA), superoxide dismutase (SOD) and glutathione peroxidase (GPX) levels using the commercial assay kits, as per manufacturer's instructions (Jiancheng Institute of Biotechnology, Nanjing, China). The protein concentrations in tissue homogenates were measured by Bradford protein assay using bovine serum albumin (BSA) as the standard (Tiangen Biotech, Beijing, China).

### Histological analysis

Liver tissues were fixed in 10% formalin and embedded in paraffin for histological assessment. Samples were subsequently sectioned (5 mm), stained with haematoxylin and eosin (H&E) and examined using bright field light microscopy (Olympus, Tokyo, Japan).

### Western blot analysis

The protein concentration was determined using the Bradford protein assay (Tiangen Biotech, Beijing, China). Proteins were resolved by SDS-PAGE and transferred to PVDF membranes (Millipore Corporation, Boston, ME, USA). After blocking in TBST containing 5% skimmed milk powder, the membranes were incubated overnight at 4°C with primary antibodies against cleaved CYP2E1 (1:500; Boster, Wuhan, China) and glyceraldehyde phosphate ehydrogenase (ß-actin, 1:1000; Tianjin Sungene Biotech, Tianjin, China). Blots were then incubated with a 1:2000 dilution of horseradish peroxide conjugated secondary antibodies (Sungene Biotech, Tianjin, China) for 2 h at room temperature. Protein bands were visualised by ECL reaction (Genshare Biological, Xi'an, China). The protein levels were quantified using Gel-Pro Analyzer software (Media Cybernetics, Washington, MD, USA).

### Determination of the inflammation-related gene and antioxidant factor expressions by real-time quantitative PCR

The mRNA was isolated from the frozen liver tissues using a Total RNA isolation kit (Tiangen Biotech, Beijing, China), according to the manufacturer's instructions. The mRNA samples were then reverse-transcribed into cDNA using HiScript™ RT SuperMix for Real-Time Polymerase Chain Reaction (qPCR; Vazyme Biotech, Nanjing, China). The aliquots of cDNAs were amplified using specific primers (Table 1). The real-time PCR was performed on a QuantStudio^TM^ 6 Flex Real-Time PCR System (Life Technologies, MD, USA) with SYBR^®^ Green Master Mix (Vazyme Biotech, Nanjing, China). The relative expression of mRNA was expressed by the 2 ^-(ΔΔCt)^ formula and normalised to that of β-actin, an internal control gene.

### Statistical analysis

All the experimental data were expressed as mean ± SD. The significant difference from the respective control in all experiments was assessed by one-way analysis of variance (ANOVA) using SPSS (IBM Corporation, Chicago, USA). Values of *p*< 0.05 were considered statistically significant.

## Results

### Pharmacokinetic analysis of APAP and ROX

**Figure 1:**
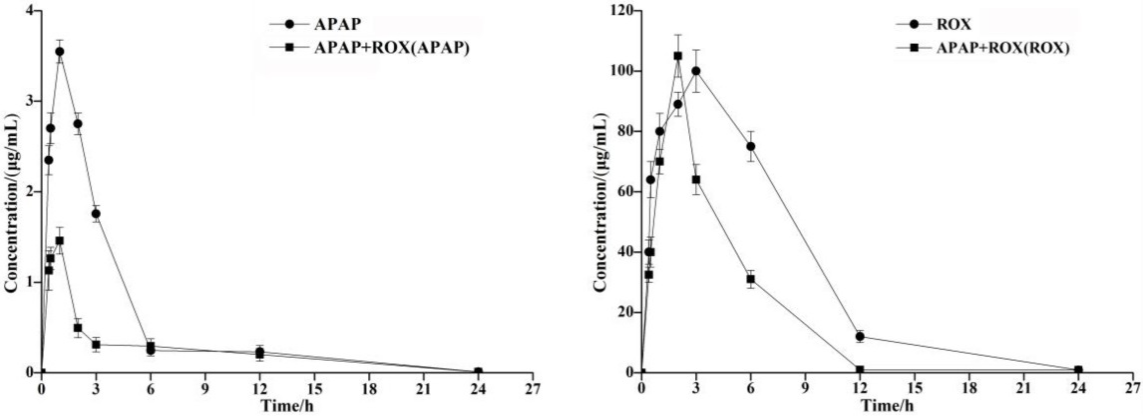
**Time-concentration curves of APAP and ROX in rats.** The AUC of APAP and ROX co-treatments was significantly reduced compared to either APAP or ROX alone.

The concentration of the APAP and ROX standard solution (μg/mL) was plotted on the abscissa. The standard working curve of APAP as Y=135.53x+6.5144 (R^2^=0.999) and of ROX as Y=3.9328x+21.97 (R^2^=0.992) provided peak areas of each component to be plotted on the ordinate. The pharmacokinetic parameters of APAP and ROX were determined by HPLC, and the C_max_ of the APAP and ROX co-treatment was significantly increased compared to the separated APAP or ROX treatment (Table 2). The AUC of APAP and ROX co-treatment was significantly reduced compared to either APAP or ROX alone (Figure 1).

### Co-treatment effects of APAP and ROX on CYP2D6 and CYP2E1

The standard solution concentration (μg/mL) of the cocktail substrate (chlorzoxazone and dextromethorphan) was taken as the abscissa. The standard working curves of chlorzoxazone as Y=19.328x+18.6 (R^2^=0.999) and dextromethorphan as Y=25.927x-9.1585 (R^2^=0.99) were both plotted on the ordinate.

**Figure 2:**
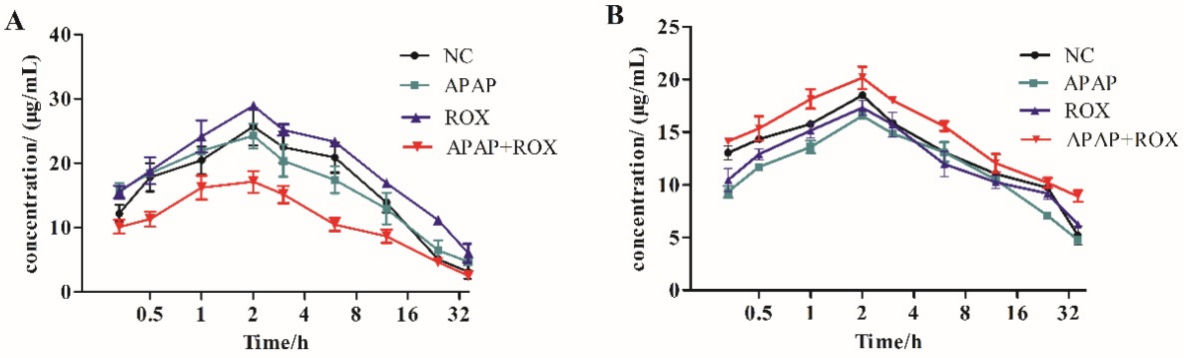
** Time-concentration curves.** Substrate inductions: (A) chlorzoxazone; (B) dextromethorphan; mean + SD (n=4). The co-treatment of APAP and ROX had different effects on the CYP450 subtypes, which induced CYP2E1 and inhibited CYP2D6.

The pharmacokinetic effect of CYP450 was compared with the cocktail probe drug method in the APAP and ROX co-treatment group. The activity of CYP2E1 and CYP2D6 accelerated the metabolism of chlorzoxazone and decelerated that of dextromethorphan, respectively. The co-treatment of APAP and ROX had different effects on the CYP450 subtypes, namely inducing CYP2E1 and inhibiting CYP2D6 (Figure 2).

### Co-treatment of APAP and ROX on hepatic dysfunction

**Figure 3:**
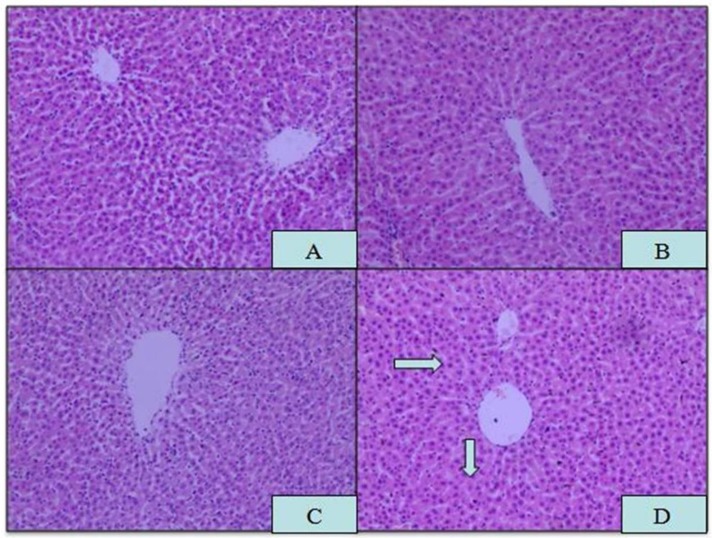
**Histopathological changes in liver tissue.** Magnification: 10×10; (A) NC; (B) APAP; (C) ROX; (D) APAP-ROX. →Dissolution of the nucleus. The liver cells showed pathological changes in loose arrangement, extravasated blood and vascular degeneration (D).

**Figure 4:**
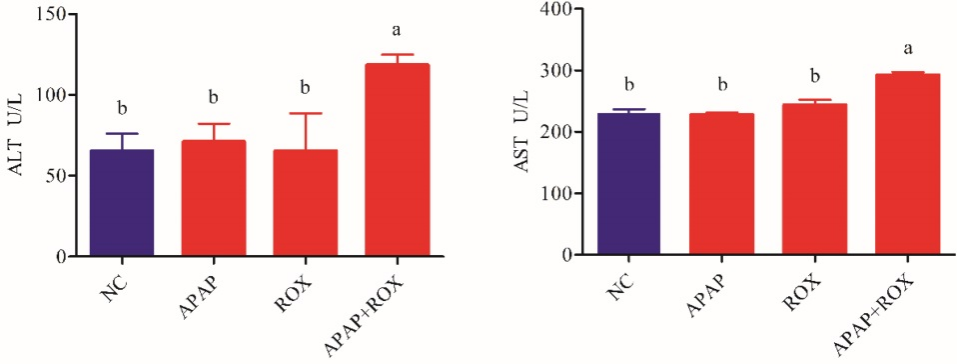
** Increased ALT and AST in APAP-ROX co-treatment.** Each value represents the mean ± SD of three independent experiments; labels a-d indicate statistically different groups (*p*< 0.05). The serum ALT and AST activities of APAP-ROX co-treatment groups were significantly increased in the co-treatment group compared with the NC group.

Microscopic analysis of tissue sections of rats from the APAP, ROX and APAP-ROX co-treatment groups revealed considerable changes from co-treatment (Figure 3). The liver cells showed pathological changes in loose arrangement, extravasated blood and vascular degeneration (Figure 3D); further, serum ALT and AST activities were significantly increased in the co-treatment group compared with the NC group (Figure 4).

### Co-treatment of APAP and ROX on liver oxidative stress

The co-administration of APAP-ROX resulted in significantly increased levels of MDA and depleted GPX and SOD activity levels. Simultaneously, the chemokine mRNA levels were analysed by Q-RTPCR. The levels of Nrf-2, GSTA, GCLC-1, HO-1 and NQO1 had no significant differences in the APAP group and ROX group compared with the NC group (*p*> 0.05; Figure 6), suggesting that a single administration of APAP and ROX did not induce inflammation. In contrast, in the co-treatment group, the expression of those chemokine mRNA markedly increased compared to the NC group (*p*< 0.05), indicating that co-treatment of APAP and ROX induced liver injury and increased the activities of Nrf-2, GSTA-2, GCLC-1, HO-1 and NQO1 (Figure 6).

### Determination of CYP2E1 expressions on APAP and ROX

The expressions of the CYP2E1 protein were markedly increased in the APAP and ROX groups (*p*< 0.05), particularly in the co-treatment group, when compared to the NC group (*p* < 0.01; Figure 7).

### The effect of APAP and ROX on liver inflammation

The levels of TNF-α, INF-γ, VCAM-1, CXCL-1 and STAT-3 were not significantly different in the APAP group and ROX group expression levels compared with the NC group. The levels of hepatic TNF-α, INF-γ, VCAM-1, CXCL-1 and STAT-3 significantly increased co-treatment of the APAP and ROX groups compared with the NC group (*p*< 0.05; Figure 8).

## Discussion

Drug-drug interactions are usually unfavourable. DDIs affect the metabolism of each drug in the body, which may change CYP450 enzyme activity. In normal conditions, APAP is predominantly metabolised in the liver by conjugating with glucuronic acid and sulphate 9. Overdosed APAP is metabolised by CYP2E1, which catalyses two-electron oxidation to reactive and toxic N-acetyl-p-benzoquinone imine (NAPQI) and induces oxidative stress, mitochondrial dysfunction, inflammation and DNA damage 10. The hepatotoxicity induced by APAP exhibited a circadian rhythm with the peak liver toxicity when administrated (injection) at evening (20:00), while with markedly decreased liver damage when the administration is conducted at morning (08:00). Furthermore, the circadian rhythms may be associated with the expression of hepatic glutathione (GSH) 11 under the mediation of clock gene such as mPer2 12. In the current study, the APAP and ROX combination resulted in prolonged clearance of APAP (Table 2) and enhanced APAP toxicity.

Cellular CYP2E1 is known to mediate prolonged alcohol and APAP induced toxicity in hepatic and extra-hepatic cells. Chronic ethanol intake may enhance APAP toxicity by producing a persistent up-regulation of CYP2E1, as well as depleting GSH stores 13, 14. In the current study, APAP and ROX were administrated to rats, with the dose calculated to be standard through conversion of human body surface area to rat body surface area. The APAP-ROX co-treatment increased CYP2E1 activity and decreased CYP2D6 activity in rat hepatocytes (Figure 2). Consequently, the combined management of APAP-ROX resulted in the long-term clearance of APAP and ROX (Table 2).

Clinically, the activities of AST and ALT are regarded as sensitive indicators of hepatotoxicity 15. The hepatoxicity was observed in the rat after APAP and ROX co-treatment, as characterised by higher levels of serum ALT, AST and hepatic histopathological lesions.

**Figure 5:**
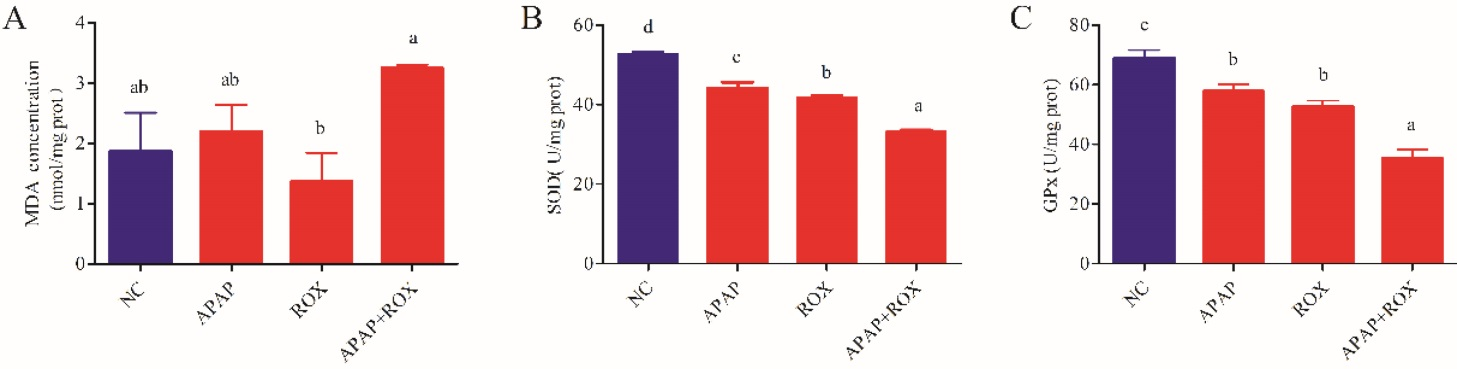
**The oxidative changes in liver tissue.** Notes: Each value represents the mean ± SD of three independent experiments; labels a-d indicate statistically different groups (*p*< 0.05). The APAP and ROX co-treatment increased the MDA level and depressed GPX and SOD activities.

**Figure 6:**
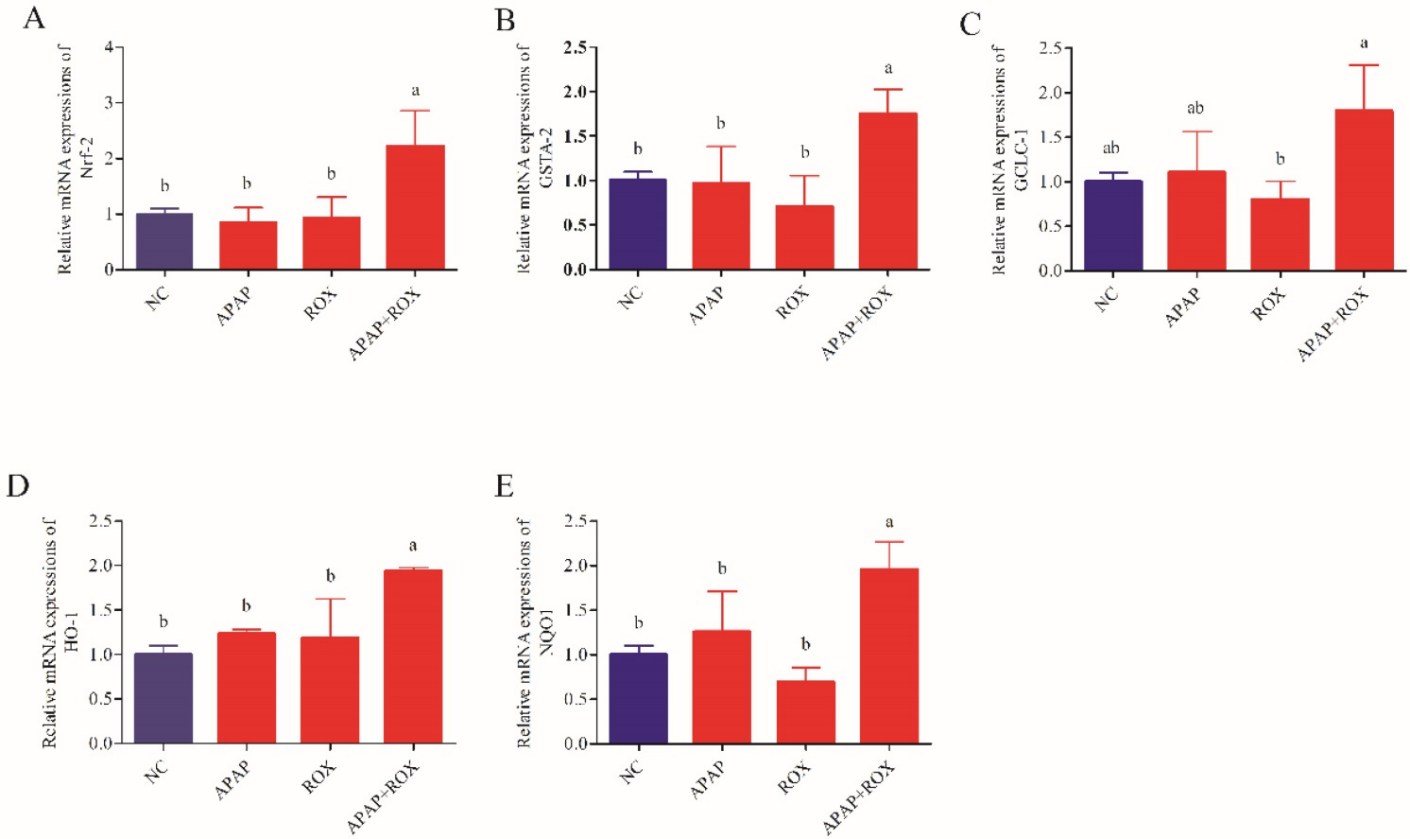
**Effect of APAP-ROX on the chemokine mRNA expression levels.** Each value represents the mean ± SD of three independent experiments; labels a-d indicate statistically different groups (*p*< 0.05). The levels of Nrf-2, GSTA, GCLC-1, HO-1 and NQO1 had no significant differences in the APAP group and ROX group compared with the NC group. In the co-treatment group, the expression of those chemokine mRNA markedly increased compared to the NC group.

**Figure 7:**
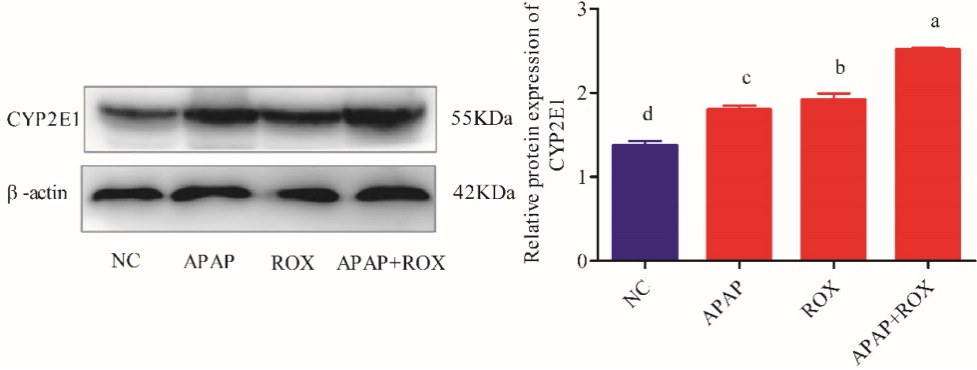
** Liver CYP2E1 expression.** Each value represents the mean ± SD of three independent experiments; labels a-d indicate statistically different groups (*p*< 0.05). The expressions of the CYP2E1 protein were markedly increased in the APAP and ROX groups (*p*< 0.05), particularly in the co-treatment group, when compared to the NC group.

**Figure 8:**
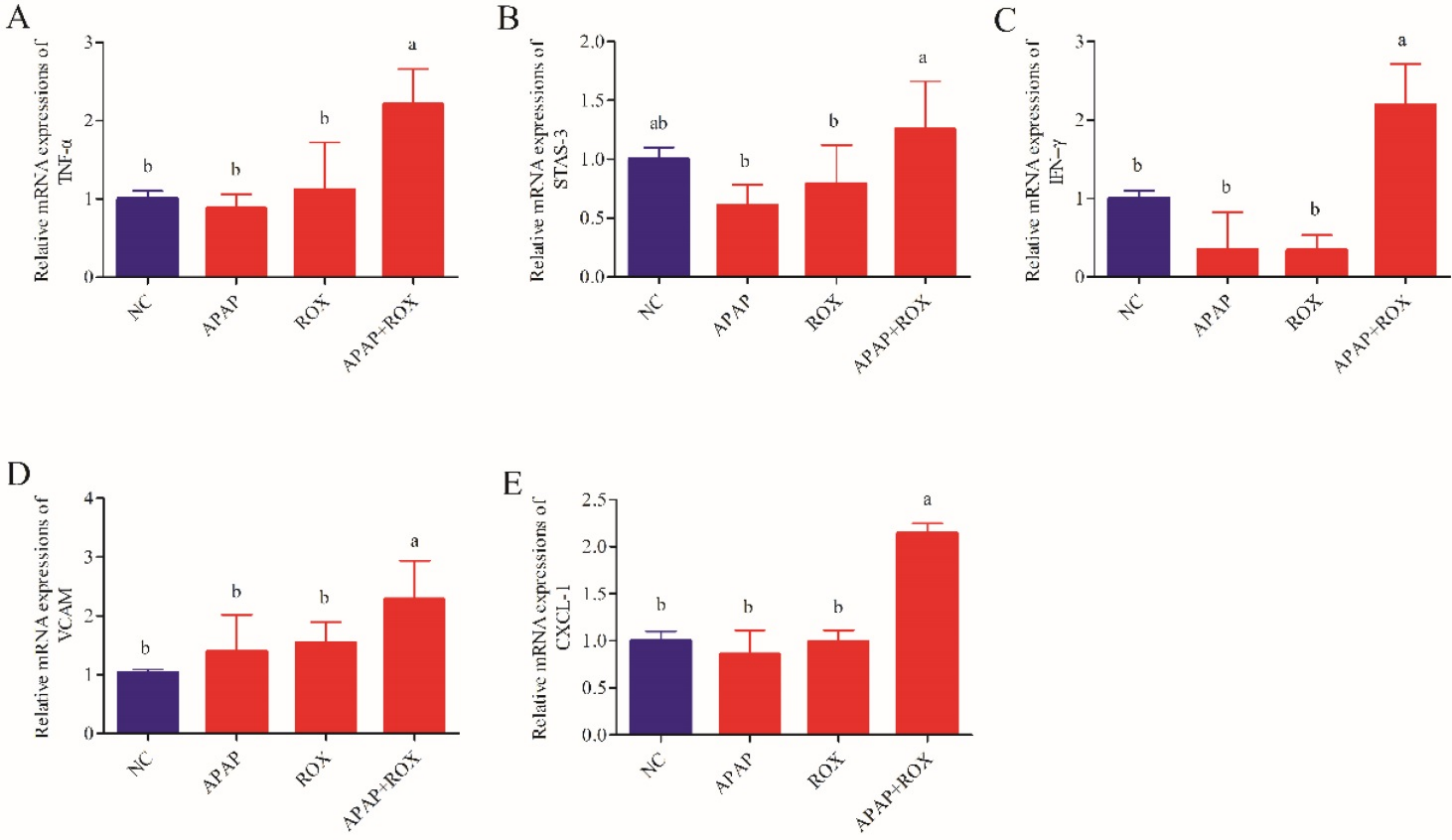
**The expression level of inflammation factors mRNA.** Each value represents the mean ± SD of three independent experiments; labels a-d indicate statistically different groups (*p*< 0.05). The levels of hepatic TNF-α, INF-γ, VCAM-1, CXCL-1 and STAT-3 significantly increased co-treatment of the APAP and ROX groups compared with the NC group.

Healthy human cells have an effective anti-oxidative and anti-inflammatory defence system, in which SOD and GPX are the main enzymes 16. When drug intake is excessive, the body's scavenging capacity changes, and oxidative damage occurs in the liver 17. The current study confirmed that an APAP and ROX co-treatment increased the MDA level and depressed GPX and SOD activities (Figure 5), suggesting the impairment of hepatic redox homeostasis, accumulation of ROS and formation of lipid peroxidation. Co-treatment significantly increased the mRNA expression of Nrf2 (Figure 6), which is significant because the activated Nrf2 is a transcription factor that modulates endogenous antioxidant enzymes 18, 19. APAP and ROX stimulate Nrf2 activation, which binds to the antioxidant response element and further activates the transcription of gene-encoding for antioxidants and detoxification, including haem oxygenase-1 (HO-1), NADPH, quinone oxidoreductase-1 (NQO-1) and glutathione-synthesising enzymes glutamate-cysteine ligase catalytic subunit (GCLC). The results suggest that increased mRNA expression of Nrf2 after co-treatment can lead to transcriptional activation of antioxidant enzymes (HO-1, SOD and CAT; Figure 6).

Drug candidates that cause intrinsic liver injury are usually weeded out in preclinical testing, the intrinsic reaction of APAP are predictable, dose-dependent, and usually occur in overdose settings or with pre-existing hepatic impairment. Idiosyncratic drug-induced liver injury could be widely existed among pharmaceutical products, but with limited confirmed information. It is interesting and necessary to further investigate whether ROX may lead to an idiosyncratic reaction, and whether it is dosage, patient or environmental risk related 20, 21, especially in the content of drug-drug interactions. ROS-mediated inflammation plays a vital role in the pathogenesis of APAP 22. During the inflammation caused by APAP and ROX overdose, the cytokines were up-regulated and accumulated in the liver. Among these, TNF-α and Nrf-2 have been recently implicated as critical mediators of APAP-induced hepatotoxicity 23, 24. Our study demonstrated that APAP and ROX co-treatment significantly increased the expression levels of TNF-α, INF-γ, VCAM-1, CXCL-1, STAT-3, Nrf-2, GSTA, GCLC-1, HO-1 and NQO1, suggesting that APAP and ROX co-treatment can induce liver inflammation.

In summary, the combination of APAP and ROX caused metabolic changes in each drug and led to a certain degree of liver damage, as co-treatment inhibited the activities of CYP2D6 and increased the expressions and activities of CYP2E1, which led to slower APAP eliminations and higher drug plasma levels, thereby inducing oxidative stress and hepatotoxicity.

## Figures and Tables

**Table 1:** Primers used for real-time quantitative PCR

Target gene	Forward primer (5'-3')	Reverse primer (5'-3')
TNF-α	TATGGCTCAGGGTCCAACTC	GCTCCAGTGAATTCGGAAAG
IFN-γ	TCAAGTGGCATAGATGTGGAAGAA	TGGCTCTGCAGGATTTTCATG
VCAM-1	AGCCTCAACGGTACTTTGGA	GCGTTTAGTGGGCTGTCTAT
CXCL-1	GATTCACCTCAAGAACATCCAGAG	GAAGCCAGCGTTCACCAGAC
STAT-3	TGCAGAGCAGGTATCTTGAG	TGCTGCTTCTCTGTCACTAC
Nrf-2	GCTGATGGAGTACCCTGAGGCTAT	ATGTCCGCAATGGAGGAGAAGTCT
GSTA-2	TCAGTAACCTGCCCACAGTGAAGA	GCATGTTCTTGACCTCTATGGCTGG
GCLC-1	TGAGATTTAAGCCCCCTCCT	TTGGGATCAGTCCAGGAAAC
HO-1	TGCCAGTGCCACCAAGTTCAAG	TGTTGAGCAGGAACGCAGTCTTG
NQO1	GGAGACAGCCTCTTACTTGCCAAG	CCAGCCGTCAGCTATTGTGGATAC
β-actin	CGTTGACATCCGTAAAGACCTC	TAGGAGCCAGGGCAGTAATCT

**Table 2:** The pharmacokinetic parameters of APAP and ROX

Parameter	APAP	Co-treatment	ROX	Co-treatment
T_1/2 z_ (h)	4.74 ±1.00	19.10 ± 0.63*	4.07± 3.20	3.05± 2.56
T_max_ (h)	0.56 ± 0.41	1.00 ± 0.26	3.00± 0.88	1.00± 0.37^#^
C_max_ (mg/L)	3.91 ± 0.99	3.44 ± 0.68	93.64± 52.56	64.12 ± 42.75^#^
MRT (0-∞) (h)	5.97 ± 1.10	20.64 ±1.21	5.80 ± 4.78	4.99 ± 3.21
AUC (0-∞) (mg/h/L)	27.11± 4.00	15.45±2.63*	796.37 ± 452.68	391.64± 83.41^#^
CLz (L/h/kg)	1.05 ± 0.43	1.294 ± 0.32	0.025± 0.040	0.051± 0.061

Note: *refers to the significant difference of APAP group to control (*p*<0.05); # refers to the significant difference of ROX group to control (*p*<0.05).
